# Targeted massively parallel sequencing of angiosarcomas reveals frequent activation of the mitogen activated protein kinase pathway

**DOI:** 10.18632/oncotarget.5936

**Published:** 2015-09-30

**Authors:** Rajmohan Murali, Raghu Chandramohan, Inga Möller, Simone L. Scholz, Michael Berger, Kety Huberman, Agnes Viale, Mono Pirun, Nicholas D. Socci, Nancy Bouvier, Sebastian Bauer, Monika Artl, Bastian Schilling, Tobias Schimming, Antje Sucker, Benjamin Schwindenhammer, Florian Grabellus, Michael R. Speicher, Jörg Schaller, Uwe Hillen, Dirk Schadendorf, Thomas Mentzel, Donavan T. Cheng, Thomas Wiesner, Klaus G. Griewank

**Affiliations:** ^1^ Department of Pathology, Memorial Sloan Kettering Cancer Center, New York NY, USA; ^2^ Marie-Josée and Henry R. Kravis Center for Molecular Oncology, Memorial Sloan Kettering Cancer Center, New York NY, USA; ^3^ Human Oncology and Pathogenesis Program Memorial Sloan Kettering Cancer Center, New York NY, USA; ^4^ Department of Dermatology, UUniversity Hospital Essen, University of Duisburg-Essen, Essen, Germany and German Cancer Consortium (DKTK), Heidelberg, Germany; ^5^ Department of Ophthalmology, University Hospital Essen, University of Duisburg-Essen, Essen, Germany and German Cancer Consortium (DKTK), Heidelberg, Germany; ^6^ Department of Medical Oncology, University Hospital Essen, University of Duisburg-Essen, Essen, Germany and German Cancer Consortium (DKTK), Heidelberg, Germany; ^7^ Institute of Pathology, University Hospital Essen, West German Cancer Center, University Duisburg-Essen and the German Cancer Consortium (DKTK), Essen, Germany; ^8^ Institute of Human Genetics, Medical University of Graz, Graz, Austria; ^9^ Department of Dermatology, Medical University of Graz, Graz, Austria; ^10^ Dermatopathology Duisburg, Duisburg, Germany; ^11^ Dermatopathology Friedrichshafen, Friedrichshafen, Germany

**Keywords:** angiosarcoma, genetics, MAPK pathway, MYC

## Abstract

Angiosarcomas are rare malignant mesenchymal tumors of endothelial differentiation. The clinical behavior is usually aggressive and the prognosis for patients with advanced disease is poor with no effective therapies. The genetic bases of these tumors have been partially revealed in recent studies reporting genetic alterations such as amplifications of *MYC* (primarily in radiation-associated angiosarcomas), inactivating mutations in *PTPRB* and R707Q hotspot mutations of *PLCG1*. Here, we performed a comprehensive genomic analysis of 34 angiosarcomas using a clinically-approved, hybridization-based targeted next-generation sequencing assay for 341 well-established oncogenes and tumor suppressor genes. Over half of the angiosarcomas (*n* = 18, 53%) harbored genetic alterations affecting the MAPK pathway, involving mutations in *KRAS*, *HRAS*, *NRAS*, *BRAF*, *MAPK1* and *NF1*, or amplifications in *MAPK1*/*CRKL*, *CRAF* or *BRAF*. The most frequently detected genetic aberrations were mutations in *TP53* in 12 tumors (35%) and losses of *CDKN2A* in 9 tumors (26%). *MYC* amplifications were generally mutually exclusive of *TP53* alterations and *CDKN2A* loss and were identified in 8 tumors (24%), most of which (*n* = 7, 88%) arose post-irradiation. Previously reported mutations in *PTPRB* (*n* = 10, 29%) and one (3%) *PLCG1* R707Q mutation were also identified. Our results demonstrate that angiosarcomas are a genetically heterogeneous group of tumors, harboring a wide range of genetic alterations. The high frequency of genetic events affecting the MAPK pathway suggests that targeted therapies inhibiting MAPK signaling may be promising therapeutic avenues in patients with advanced angiosarcomas.

## INTRODUCTION

Angiosarcomas are rare malignant mesenchymal tumors of endothelial differentiation [1]. Risk factors for their development include radiation, chronic lymphedema (e.g. Stewart-Trewes Syndrome) and exogenous toxins (e.g. vinyl chlorides, arsenic, etc.). Solar ultraviolet radiation (UV)-exposure is assumed to be a pathogenetic factor for cutaneous tumors which most frequently arise in the head and neck region in the absence of other risk factors. The breast is the most frequent location in which radiation- and lymphedema-associated angiosarcomas arise. Prognosis is poor with a 5-year overall survival of 35% and a median overall survival of 7 months [2]. Treatment with targeted agents such as sorafenib [3-5] and bevacizumab [6] as well as pazopanib [7, 8] has led to transient partial responses or stable disease in selected patients. Clinical trials are currently evaluating tyrosine kinase inhibitors with anti-angiogenic properties including pazopanib (e.g. NCT01462630, NCT02212015, etc.), axitinib (NCT01140737) and regorafenib (NCT02048722). Despite these attempts however, the overall prognosis for patients with advanced angiosarcoma remains poor, and novel mechanism-based, effective therapeutic modalities are needed.

The genetic alterations responsible for the pathogenesis of angiosarcomas are incompletely understood. Whereas characteristic translocations have been identified in many types of sarcoma, none have been described to date in angiosarcomas [9-11]. Amplification of *MYC* has been reported in a high percentage of post-radiation angiosarcomas (55-100%)[12-14], being considerably less frequent in angiosarcomas arising in the absence of prior radiation therapy [12, 15]. Amplification of *FLT4* (18-25%) and *KDR* mutations (~10%) have also been described [14, 16, 17]. A recent publication described recurrent R707Q hotspot mutations in the *PLCG1* gene (3 of 34 tumors, 9%) as well as mutations in *PTPRB* [18] (10 of 39 tumors, 26%). Recurrent mutations in *PLCG1* R707Q in angiosarcomas were confirmed in a subsequent study [19] (3 of 10 tumors, 30%).

To identify potentially novel genetic alterations that play a role in the pathogenesis and to better understand the distribution of known genetic alterations, we comprehensively genotyped a cohort of 34 angiosarcomas. We used a clinically-approved, hybridization-based next-generation sequencing assay targeting 341 known oncogenes and tumor suppressor genes [20] and additionally sequenced *PLCG1* and *PTPRB*.

## RESULTS

### Tumors and patients

Tumor samples were obtained from 34 individuals (24 women, 10 men), with a median age of 69 (range 25-85) years. Angiosarcomas arose in skin (*n* = 22), liver (*n* = 4), spleen, kidney, adrenal, thyroid, nasal cavity, lymph node and mediastinum (*n* = 1 each). In one case the origin was unknown. The samples were primary tumors in 26 cases, 3 were a recurrence, 4 were metastases and in 1 case the sample type was not known. FNCLCC (Fédération Nationale des Centres de Lutte Contre le Cancer) grades were: grade 1 (*n* = 1), grade 2 (*n* = 14) and grade 3 (*n* = 18). Seven tumors developed following radiation therapy, and 9 tumors arose on the head and neck in areas with considerable cumulative sun exposure.

### Genomic alterations (mutations and copy number variations)

Median Sequencing coverage exceeded 165x in 31 tumors. In 3 cases, the median coverage was < 100x, but still allowed identification of hotspot mutations.

### MAPK pathway alterations

**Figure 1:**
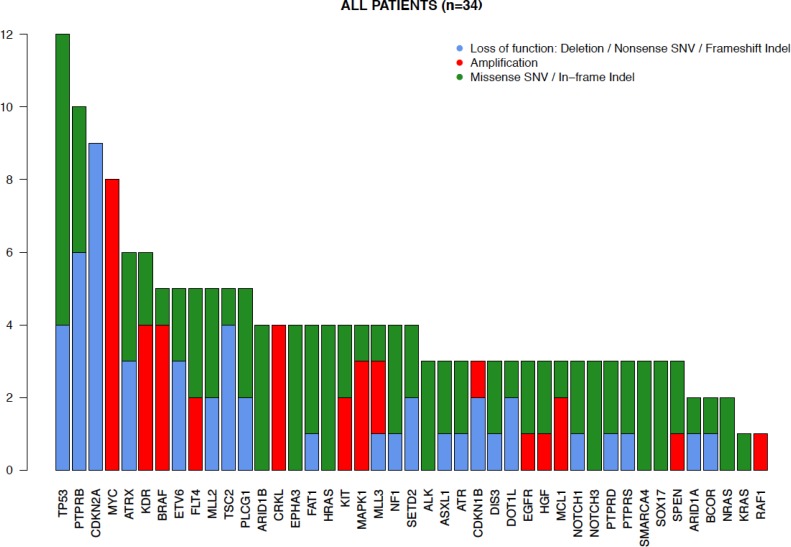
Distribution of genetic alterations in angiosarcomas The gene alterations identified in the 34 tumors analyzed are demonstrated in the bar graph. The y-axis depicts the amount of tumor samples harboring the alterations. Loss-of-function (deletion, nonsense single nucleotide variations (SNV) and frameshift mutations or indels) alterations are shown in blue, amplifications in red, and missense SNV or in-frame indels (insertions or deletions) in green.

Alterations involving the MAPK pathway were identified in 18 (53%) tumors. In 9 tumors, hotspot mutations in *KRAS* (G12), *HRAS* (A59, Q61), *NRAS* (Q61), *BRAF* (V600) and *MAPK1* (E322) were observed. CNAs, including focal amplifications in *MAPK1*/*CRKL* (chr22q11), *CRAF* (chr3p25) or broad chromosomal gains on chr7 including *BRAF* were seen in 8 tumors (Figures 1 + 2). One tumor showed an inactivating *NF1* intragenic deletion as a mechanism for activating the MAPK pathway. The tumor positive for a *KRAS* G12D mutation also harbored a known activating *GNAQ* R183Q mutation, albeit at varying allelic frequencies (*KRAS* G12D at 6%, *GNAQ* R183Q at 30%), suggesting that the sample may be heterogeneous and that the *KRAS* mutant allele may be present in a sub-clone. With the exception of the tumor with *GNAQ* and *KRAS* co-mutation, all MAPK-activating hotspot mutations appeared to be mutually exclusive of each other and amplifications of MAPK pathway genes (Figure 3).

**Figure 2:**
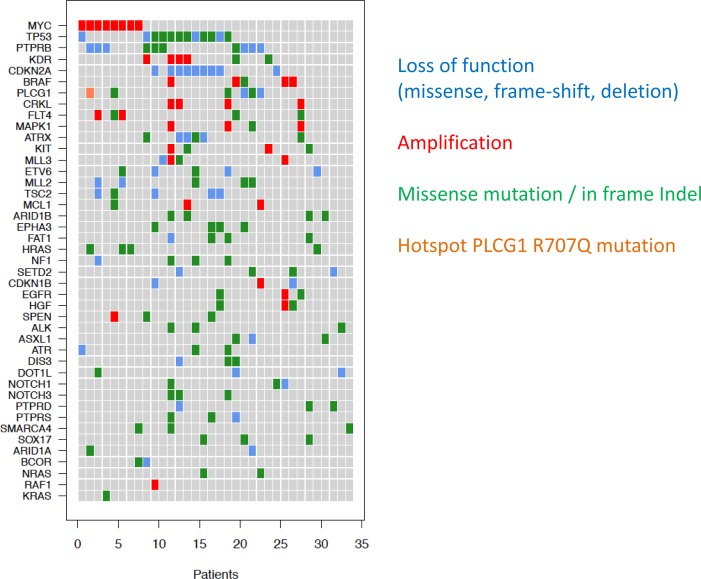
Co-occurence of mutations in angiosarcoma samples Recurrently mutated genes are shown on the y-axis and the samples (patients) on the x-axis. Loss-of-function mutations (missense, frame-shift, deletion) are shown in blue, amplifications in red, missense/in frame indels (insertions or deletions) in green and the hotspot *PLCG1* R707Q mutation in orange.

**Figure 3:**
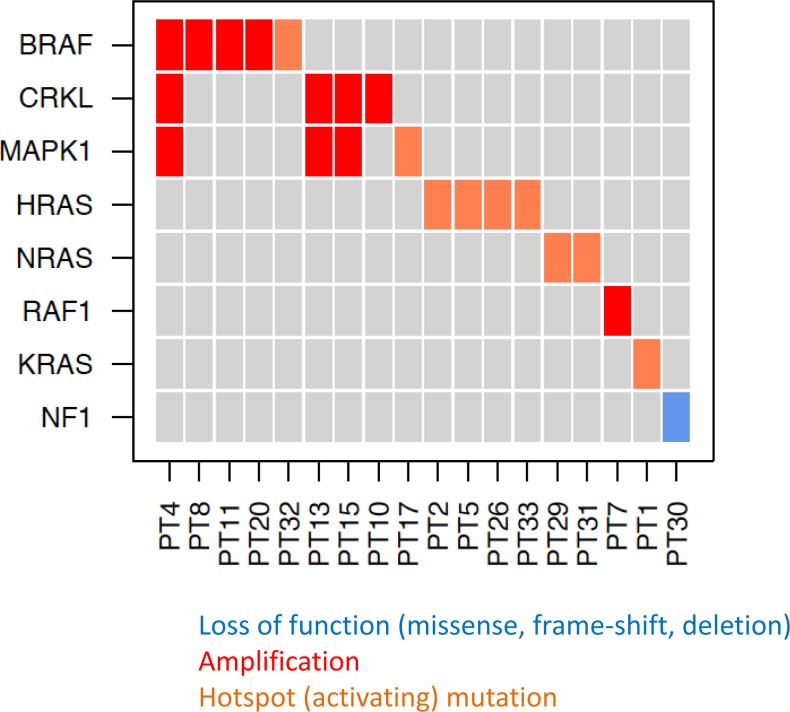
Genetic alterations in angiosarcomas affecting the MAPK pathway Loss-of-function mutations (missense, frame-shift, deletion) are shown in blue, amplifications in red, activating mutations in orange.

### Additional gene mutations (non-MAPK)

*TP53* mutations were the most frequently identified mutations (*n* = 12, 35%). Virtually all *TP53* mutations (*n* = 11, 92%) were in samples lacking *MYC* amplifications. *PTPRB* mutations were identified in 10 samples, and 6 (60% of the mutations, identified in 20% of the tumors) of these were clearly inactivating (nonsense or frameshift) mutations. The putatively activating hotspot mutation in *PLCG1* (R707) was observed in one (3%) tumor. Additional loss-of-function mutations were detected in *ATRX* (*n* = 4), *MLL2* (*n* = 2) and *ASXL1* (*n* = 1) (Figure 1). Two missense mutations in *KDR* were identified (Table 1).

**Table 1:** Clinical features and key genetic alterations

Case no.	Sex	Age	Site	Tumor type	Associations	Key genetic events	*MYC* amp	*CDKN2A* loss
1	F	71	Trunk	Primary	RTX	*GNAQ* c.547G>A. R183Q; *KRAS* c.35G>A, p.G12D; *PTPRB* c.6409C>T, p.Arg2137*	Present	-
2	F	79	Trunk - left axilla	Primary	RTX	PLCG1 c.2120G>A, p.Arg707Gln; *PTPRB* c.3085G>T, p.Glu1029*; *HRAS* c.175G>A, p.A59T	Present	-
3	F	46	Trunk - vulva	Primary	CSD		-	Present
4	M	72	HN - eyelid	Primary	CSD	*BRAF* amp; *CRKL* amp; *MAPK1* amp; *KDR* amp	-	Present
5	F	68	Trunk	Recurrence	RTX	*HRAS* c.175G>A, p.A59T, FLT4 amp	Present	-
6	F	74	HN - nasal cavity	Primary			-	-
7	M	84	HN - right temporal	Recurrence	CSD	*RAF1* amp	-	Present
8	F	30	Mediastinum	Primary		*BRAF* amp, *KDR* c.2159G>C, p.R720P	-	-
9	F	61	HN - thyroid	Primary			-	-
10	F	75	HN	Primary	CSD	*CRKL* amp; *PTPRB* c.5713_5714insT, p.Gln1905fs; *KDR* amp	-	Present
11	F	73	Lower limb - foot	Primary	CSD	*BRAF* amp	-	-
12	F	49	Not known (cutaneous)	Not known	CSD		-	-
13	F	77	HN - left neck	Primary		*CRKL* amp; *MAPK1* amp	-	-
14	M	48	Lung (primary kidney)	Metastasis		*KDR* c.2312C>A, p.T771K	-	-
15	M	70	HN - right supraclavicular	Primary		*CRKL* amp; *MAPK1* amp	-	-
16	F	81	Liver	Primary		*KDR* amp	-	-
17	M	42	Lung (primary unknown)	Metastasis		*MAPK1* c.964G>A, p.E322K	-	-
18	F	74	HN	Primary	CSD		-	Present
19	M	68	HN - orbicularis oculi	Recurrence			-	Present
20	F	63	Lower limb - lateral malleolus	Primary		BRAF amp	-	-
21	F	61	Adrenal gland	Primary			-	-
22	F	85	Lower limb	Primary		*PTPRB* c.6211C>T, p.Gln2071*	-	-
23	F	44	Trunk	Primary	RTX		Present	-
24	F	64	Liver	Primary		*KDR* amp	Present	-
25	M	42	Liver (primary spleen)	Metastasis			-	Present
26	F	79	HN - cheek	Primary	CSD	*HRAS* c.182A>T, p.Q61L	-	-
27	F	73	Liver	Primary			-	Present
28	M	56	Mediastinum (primary leg)	Metastasis			-	-
29	M	51	HN - nose	Primary		*NRAS* c.182A>G, p.Q61R	-	-
30	F	75	Trunk	Primary	RTX	*NF1* deletion (exon 9 and 10); *PTPRB* c.3565C>T, p.Gln1189*, *FLT4* amp	Present	-
31	M	25	Liver	Primary		*NRAS* c.182A>T, p.Q61L	-	Present
32	M	74	HN	Primary	CSD	*BRAF* c.1799T>A, p.V600E; *PTPRB* c.4311G>A, p.Trp1437*	-	-
33	F	83	Trunk	Primary	RTX	*HRAS* c.182A>T, p.Q61L	Present	-
34	F	54	Trunk	Primary	RTX		Present	-

amp = amplification; F = female; M = male; HN = head & neck region; RTX = radiation therapy; CSD = cumulative sun damage

### Copy number alterations (non-MAPK)

**Figure 4:**
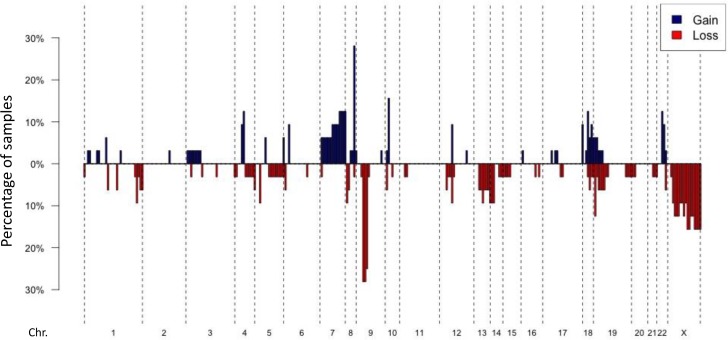
DNA copy number alterations in angiosarcomas Penetration blots of 32 angiosarcomas included in the study (two cases removed due to lower coverage). A fold change value of < −1.5 was applied as a cut off for recognizing a loss and > 1.5 for recognizing a gain. Gains are shown in blue, losses in red. The most frequent gains include *MYC* on Chr. 8, the most frequent losses *CDKN2A* on Chr. 9.

**Figure 5:**
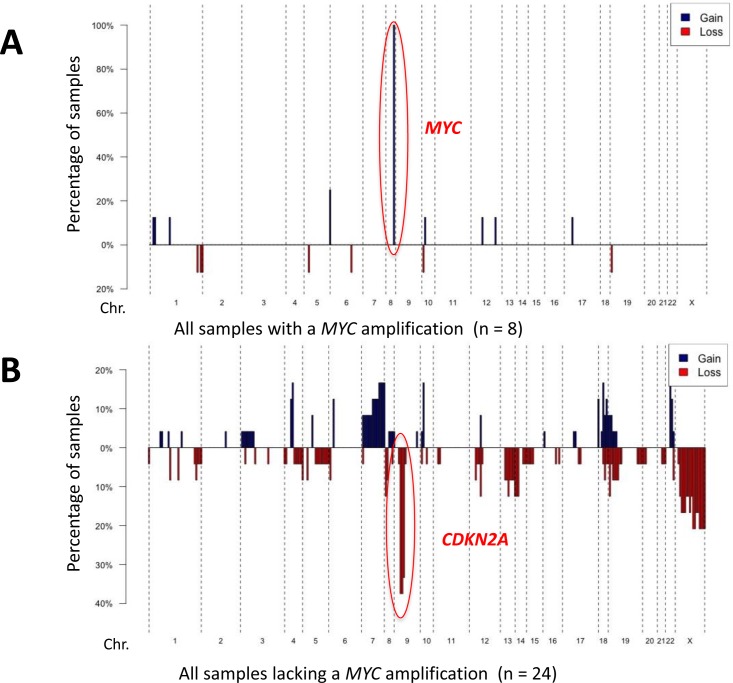
Copy number profiles according to MYC status Penetration blots of the samples included in the study grouped according to *MYC* status. **A.** penetration blot of tumors samples with a *MYC*-amplification. **B.** penetrations blot of tumors lacking a *MYC*-amplification. A fold change value of < −1.5 was applied as a cut-off for recognizing a loss and > 1.5 for recognizing a gain. Gains are shown in blue, losses in red.

Copy number information was obtained for all samples sequenced by the MSK-IMPACT assay (Figures 4 + 5). Additionally, eight samples were analyzed by array CGH, of which representative examples are shown in Supplemental Figures 1 + 2. The most frequent genetic loss (*n* = 9, 26%) affected 9p21, which includes the *CDKN2A* locus (Figures 4 + 5). *CDKN2A* losses were only found in samples arising without known prior-irradiation, all of which lacked *MYC* amplifications (Figure 5, Supplemental Figure 1).

Focal *MYC* amplifications were identified in 8 (24%) angiosarcomas*, VEGFR2* (*KDR*) amplifications in 4 (12%) tumors (Figure 1). Two samples harbored *FLT4* amplifications (Figure 5, Supplemental Figure 1). *MYC* amplifications were significantly more frequent in angiosarcomas known to be associated with previous radiation (7/7 = 100%) than in those with no history of prior irradiation (1/27 = 4%; *p* < 0.0001). Five cases with *MYC* amplifications (63%) harbored concurrent MAPK-activating mutations (Figures 2 + 4). Both samples found to have *FLT4* amplifications were in *MYC* amplified tumors arising post-radiation (Supplemental Figure 1). Aside from *MYC* amplifications, which were identified in all samples with known prior radiation therapy, *KLT4* amplifications were also found to be statistically significantly associated with previous radiation exposure (2/7 samples with previous radiation vs. 0/27 without previous radiation; *p* = 0.004).

## DISCUSSION

We identified a number of known genetic alterations in angiosarcomas, including *MYC* and *KLT4* amplifications, *RAS* mutations, inactivating *PTPRB* mutations and an activating *PLCG1* mutation. In more than half of the tumors, we found genetic alterations activating the MAPK pathway. The clinical ramifications of this finding could be significant, in that MEK inhibitors or other inhibitors of the MAPK pathway may be promising therapeutic approaches for patients with advanced angiosarcoma.

The individual genetic events activating the MAPK pathway were often mutually exclusive (Figure 3), particularly when activating hotspot mutations were present. Generally gene copy number gains are not as strongly activating as mutations leading to constitutive activation of the affected protein, which probably explains why copy number increases in MAPK genes were more frequently found to co-occur than activating mutations. This data strongly supports MAPK pathway alteration being an important common mechanism for angiosarcoma development. Presumably, strong activation of MAPK signaling allows tumor cells to continuously proliferate and, if associated with additional genetic events, transform into a malignant neoplasm. The other 47% of tumors in which no MAPK pathway activating event was identified may harbor genetic events with similar functional consequences; these events might not have been assessed in our current screen and/or may not yet be recognized as oncogenic in human malignancies.

From a therapeutic perspective, our findings provide support for targeting the MAPK pathway in many patients with angiosarcoma. In some cases, mutation-specific therapies may be available (e.g. *BRAF* V600E). In other cases in which specific inhibitors directly targeting the genetic alterations are not yet available, or no tumor-specific alteration is detected, targeting common downstream molecules of the MAPK pathway, for instance MEK with MEK inhibitors might prove to be a promising therapeutic approach. Potentially combining MAPK pathway inhibitors with other tumor-specific inhibitors, such as agents targeting *MYC* signaling (in *MYC*-amplified tumors) has the potential to significantly increase the efficacy of therapeutic regimens.

For a wide range of human malignancies, a very successful alternative therapeutic approach to small molecules with cell intrinsic inhibition of signaling pathways, has been the introduction of immune therapies in particular antibodies inhibiting PD-1 as well CTLA-4 [21-24]. Impressive response rates as well as significant numbers of long-term responses have been reported [23, 25]. Although not currently described to our knowledge, treatment with immunotherapeutic agents such as PD-1 antibodies should be considered as a possible treatment strategy for patients with advanced angiosarcomas.

We identified one activating R183Q hotspot mutation in *GNAQ*. Activating *GNAQ* mutations were originally described in melanocytic tumors and frequently occur in blue nevi, uveal melanomas [26] and central nervous system melanocytomas [27, 28]. Recently, exon 4 R183 mutations of *GNAQ* were identified in port-wine hemangionas and vascular tumors in Sturge-Weber syndrome [29]. These vascular tumors can predispose to the development of angiosarcoma. Unfortunately, in our patient harboring the *GNAQ* mutation, clinical information about an existing predisposing vascular lesion was not available. It would however be particularly intriguing for future studies to analyze if angiosarcomas arising in patients with Sturge-Weber syndrome are found to frequently harbor activating mutations in *GNAQ*. If this were the case, it could have therapeutic consequences, as approaches targeting downstream activation of *GNAQ* have shown promise in mouse models of *GNAQ*-mutated uveal melanoma [30-32] and patients with these tumors have entered clinical trials (NCT01801358). Potentially patients with angiosarcomas harboring *GNAQ* mutations could benefit from the same therapeutic approaches.

As reported in previous studies [12-14, 17], *MYC* amplifications were frequent in our cohort (8 of 34 = 24%), further supporting *MYC* copy number increase as a major activating genetic event in these tumors. *MYC* copy number increases were found in all seven tumors known to have arisen post-irradiation, strongly supporting *MYC* amplifications being a major event in this angiosarcoma subtype. However, although *MYC* clearly appears to play a very relevant role in these tumors, more than half of the *MYC*-amplified samples (5 of 8) were also found to harbor genetic alterations activating the MAPK pathway (either mutation or copy number gain). Three samples harbored Q61 *RAS* mutations, one a *NF1* deletion and one had concurrent activating *GNAQ* R183Q and *KRAS* G12D mutations. The tumor harboring a *PLCG1* R707Q mutation also harbored a *MYC* amplification. These data suggest that in addition to *MYC* amplifications, ancillary gain-of-function genetic events are necessary to enable full development of an aggressive tumor.

*FLT4* amplifications were identified in samples with previous radiotherapy at a frequency (2/7 = 29%) comparable to previous reports (18-25%) [14, 17]. The *MYC* amplification rate of 100% in tumors arising post-irradiation (7/7) we observed is in line with other studies [13, 14]. Both *KDR* mutations and amplifications occurred in tumors not known to be radiation associated (Table1, Supplemental Figure 1), with a mutation frequency (6%, *n* = 2) comparable to previous reports [16]. These data underline the alterations we observed in these genes in our tumor cohort are comparable to findings reported by other groups.

Most *TP53* mutations or deletions (11/12, 92%) and all *CDKN2A* losses occurred in tumors lacking *MYC* amplification. *TP53* mutations are frequent in UV-induced tumors and often carry a UV-signature [33, 34]; 71% of *TP53* mutations were C > T alterations, consistent with UV-induction. The distribution pattern may at least in part be explained by different pathogenic mechanisms: UV-exposure predisposing to tumors with *TP53* mutations, and radiation predisposing to angiosarcomas with *MYC* amplifications [12, 13]. Why *CDKN2A* losses were found only in non-irradiated, non-*MYC* amplified samples may be more difficult to explain, as UV-exposure is, to our knowledge, not clearly linked to DNA copy number alterations. The distribution does however clearly signify differing pathogenetic mechanisms, with tumors either prone to having *TP53* alterations and/or *CDKN2A* losses, or acquiring *MYC* amplifications. Future functional studies may reveal the reason for the relative mutual exclusivity of *MYC* amplifications with alterations of *TP53* and *CDKN2A*.

Recently described mutations affecting *PTPRB* were found in 10 samples (29%), 4 being missense and 6 inactivating, representing nonsense or frameshift mutations (Table1). As *PTPRB* consists of well over 6000 base pairs (depending on the splice variant), it is possible the relatively high frequency of mutations observed is at least partly due to the size of the gene (larger genes having a higher chance of acquiring mutations). The frequency of *PLCG1* R707Q mutations (1 of 34 = 3%) in our study was lower than in other reports, which have reported mutation frequencies ranging between 9% and 30% [18, 19]. The identification of this mutation in three studies, all leading to an identical amino acid change, indicates that these mutations are relevant pathogenic events in angiosarcoma. Overall, however, these mutations appear to be relatively uncommon, explaining only a minority of cases (pooled frequency of 9% [7 of 78 tumors] from all three studies). Studies analyzing the functional consequences of both *PTPRB* and *PLCG1* will be required before it will be possible to assess whether targeting these mutations may be a viable therapeutic approach for patients with tumors harboring these mutations.

A limitation of our study is that detailed clinical data, including therapy and follow-up information was not available. It would be interesting to see if some of the genetic alterations observed are associated with parameters such as treatment response and patient survival; however, since the genetic alterations identified are very heterogenous, such analysis would require larger sample numbers with detailed clinical follow-up data to allow a meaningful assessment of the clinical consequences of individual genetic alterations.

In summary, our study shows that angiosarcomas are pathogenetically heterogenous tumors, characterized by a range of different genetic events. Whereas the functional and clinical ramifications of some of these events, such as the recently identified *PLCG1* and *PTPRB* mutations will require further study, we found that more than half of the tumors had genetic alterations clearly linked to the MAPK signaling pathway. Almost all currently effective small inhibitor therapeutic approaches in oncology either directly or indirectly dampen the increased signaling output generated by gain-of-function genetic events. Our data showing MAPK-activating events in more than half of angiosarcomas supports the thesis that for patients with these tumors, targeting the MAPK pathway might prove to be a promising therapeutic approach.

## MATERIALS AND METHODS

### Sample selection and histopathology

Angiosarcoma tumor samples were obtained from patients treated in the Department of Dermatology and Pathology of the University Hospital Essen (21 cases), Dermatopathology Duisburg (2 cases) and Dermatopathology Friedrichshafen, Germany (11 cases). Tumor slides were reviewed by at least two experienced histopathologists (T.M., J.S., U.H., K.G.G., R.M.). Histological details are listed in Table 2. The study was done in accordance with the guidelines set forth by the ethics committee of the University of Duisburg-Essen.

**Table 2:** Pathologic features

Case no.	Growth Pattern	Cell type	Vascular structures	Cytologic atypia	TMR	Necrosis	PNI	Grade*	Immunohistochemistry		
									CD31	CD34	Other positive markers
1	Fascicles/sheets	Spindle	n.k.	Marked	13	-	-	2	+	n.k.	
2	Fascicles/sheets	Spindle/epithelioid	n.k.	Marked	8	-	-	3	+	n.k.	
3	Sheets	Primarily spindle	n.k.	Marked	13	+	-	3	+	+	ERG
4	Interspersed	Primarily spindle	Moderate	Moderate	3	-	-	2	+	n.k.	ERG
5	Fascicles/sheets	Primarily spindle	Moderate	Marked	5	+	-	3	+	+	
6	Sheets	Spindle	Moderate	Marked	3	-	-	3	+	+	
7	Fascicles	Epithelioid	Focal	Moderate	23	-	-	2	+	+	
8	Fascicles	Epithelioid	Focal	Marked	6	-	-	3	+	+	
9	Interspersed	Spindle	Moderate	Moderate	4	-	-	2	+	-	
10	Sheets	Spindle/epithelioid	Focal	Marked	18	+	+	3	+	n.k.	LIVE-1, Prox-1, podoplanin
11	Interspersed	Epithelioid	n.k.	Marked	8	-	-	3	+	n.k.	ERG
12	Sheets	Epithelioid	Absent	Marked	9	+	+	3	+	n.k.	
13	Interspersed	Spindle/epithelioid	n.k.	Moderate	6	-	-	2	+	+	
14	Interspersed	Spindle	Extensive	Mild	4	-	-	1	+	+	
15	Fascicles	Epithelioid	Focal	Moderate	9	-	-	2	+	n.k.	
16	Nodular	Epithelioid	Focal	Moderate	14	+	-	2	+	+	
17	Nodular	Spindle	Moderate	Moderate	4	-	-	2	+	+	
18	Sheets	Epithelioid	Focal	Marked	15	-	+	3	+	n.k.	ERG
19	Fascicles	Primarily spindle	Moderate	Moderate	4	-	-	n.k.	+	+	
20	Fascicles/sheets	Primarily spindle	Moderate	Marked	8	+	+	3	n.k.	+	
21	Fascicles/sheets	Epithelioid	Focal	Marked	5	-	-	3	+	+	
22	Fascicles/sheets	Primarily spindle	Moderate	Moderate	n.k.	+	-	2	+	+	
23	Nodular	Spindle	Moderate	Moderate	6	-	-	2	+	n.k.	
24	Fascicles/sheets	Spindle	Moderate	Marked	6	+	-	3	n.k.	+	
25	Sheets	Epithelioid	Focal	Marked	n.k.	-	-	3	+	n.k.	
26	Interspersed	Spindle	Moderate	Moderate	5	-	-	2	+	n.k.	
27	Interspersed	Primarily spindle	Extensive	Moderate	3	-	-	2	+	+	
28	Interspersed	Primarily spindle	Moderate	Marked	7	-	-	3	+	-	
29	Fascicles/sheets	Spindle	Focal	Marked	4	+	-	3	n.k.	n.k.	
30	Fascicles/sheets	Spindle	Moderate	Moderate	n.k.	-	+	2	+	-	
31	Fascicles	Spindle	Moderate	Marked	5	+	-	3	+	+	
32	Fascicles/sheets	Epithelioid	Moderate	Marked	12	+	+	3	+	n.k.	
33	Sheets	Epithelioid	Focal	Marked	12	+	+	3	+	n.k.	
34	Interspersed	Primarily spindle	Moderate	Moderate	7	-	-	2	+	n.k.	

TMR = tumor mitotic rate per mm^2^; Grade = histologic grade (1=low, 2=intermediate, 3=high) according to FNCLCC (Fédération Nationale des Centres de Lutte Contre le Cancer) [49]; PNI= perineural invasion; n.k.=not known (either not assessable on available material or not performed).

### DNA isolation

10μm-thick sections were cut from formalin-fixed, paraffin-embedded tumor tissues. The sections were deparaffinized and manually microdissected according to standard procedures. Genomic DNA was isolated using the QIAamp DNA Mini Kit (Qiagen, Hilden, Germany) according to the manufacturer's instructions. DNA from frozen tissue was directly applied to the Qiagen kit for purification.

### Copy number analysis

Array-based comparative genomic hybridization (CGH) was used to perform analysis of DNA copy number alterations (CNAs) in 8 cases. The methods for hybridization and analysis, including GISTIC 2.0 statistical analysis, have been described previously [35-38]. Whole genome amplification was performed using Sigma's GenomePlex^®^ Single Cell Whole Genome Amplification Kit as described previously [39].

### Hybridization-capture based next-generation sequencing for known oncogene mutations

Custom DNA probes were designed for targeted sequencing of all exons and selected introns of 341 oncogenes and tumor suppressor genes [20]. Briefly, genomic DNA from tumor samples was used to prepare barcoded libraries using the KAPA HTP protocol (Kapa Biosystems, Wilmington, MA) and the Biomek FX system (Beckman Coulter, Brea, CA). Libraries were pooled, captured and subsequently sequenced on an Illumina HiSeq 2500 system as paired-end reads. Sequenced reads were trimmed to remove vestigial adaptor sequences using TrimGalore [40], and were aligned to the hg19 human reference genome using BWA [41]. PCR duplicates were removed from the alignment output, and the aligned reads were subjected to local indel realignment and base quality recalibration using GATK [42]. Somatic variant calling was performed using MuTect [43] for single nucleotide variants (SNVs) and SomaticIndelDetector [42] for indels. Significant copy number gains and losses were detected by requiring a greater-than two-fold change in normalized coverage between tumor and a comparator reference FFPE normal. Somatic structural variants were detected using DELLY [44], requiring both paired-end and split-read support.

### *PLCG1* and *PTPRB* sequencing

A custom amplicon panel covering the *PLCG1* (75 amplicons, covering 4494 of 4613 coding bases) and *PTPRB* genes (107 amplicons, covering all 7402 coding bases) was designed and prepared applying the GeneRead Library Prep Kit from QIAGEN^®^ according to the manufacturer's instructions. NEBNext Ultra DNA Library Prep Mastermix Set and NEBNext Multiplex Oligos for Illumina from New England Biolabs were applied for adapter ligation and barcoding and 24 samples sequenced in parallel on an Illumina MiSeq. Sequence reads were aligned to the human reference sequence (hg19) using BWA and post-processed using tools from GATK and Picard for indel realignment and base quality recalibration. Variant calling for SNVs and indels was performed using a combination of MuTect [43] and Varscan [45]. Single nucleotide polymorphisms (SNPs) corresponding to common population variants were filtered out by removing variants with greater than 1% prevalence in the 1000 genomes cohort. Mutations were reported if they were predicted to result in non-synonymous alterations in protein-coding DNA sequences, and if the overall coverage of the mutation site was ≥50 reads, with ≥20 mutant reads and the variant allele frequency was ≥5%. The criteria for reporting known oncogenic hotspots from COSMIC [46] or TCGA [47, 48] was less stringent: ≥10 mutant reads and variant allele frequency ≥2%.

## SUPPLEMENTARY MATERIAL FIGURES AND TABLES


