# Supplementary material for: Targeted massively parallel sequencing of angiosarcomas reveals frequent activation of the mitogen activated protein kinase pathway
Source: Oncotarget. 2015 Sep 30;6(34):36041–52. doi: 10.18632/oncotarget.5936 (PMC4742160; doi:10.18632/oncotarget.5936)
Supplement: Supplementary file 1 [file oncotarget-06-36041-s001.pdf]

Targeted massively parallel sequencing of angiosarcomas reveals frequent activation of the mitogen activated protein kinase pathway

Supplementary Material

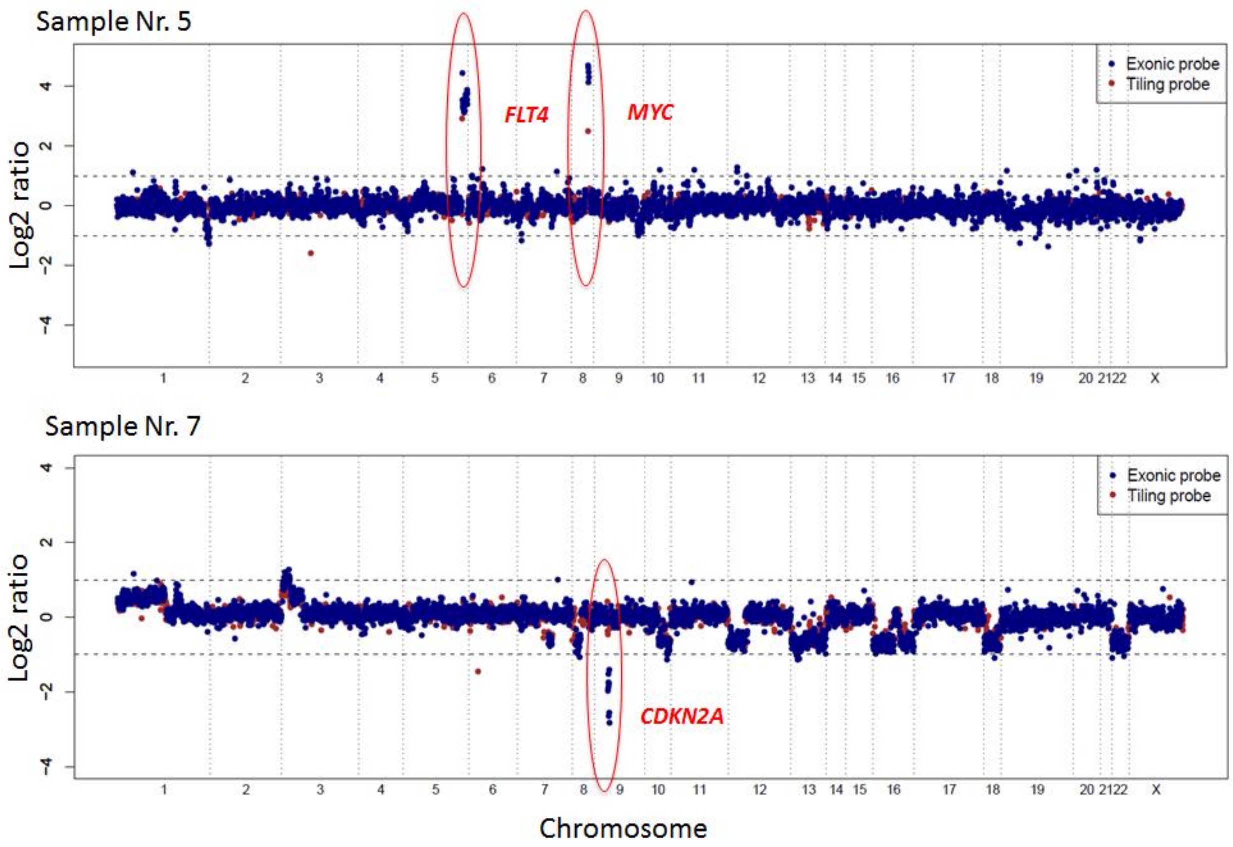

Supplemental Figure 1. Representative individual copy number profiles.

Shown on top is sample 5, a tumor known to have arisen post-irradiation, demonstrating a high level *MYC* amplification (Chr.8q) as well as a concurrent *FLT4* amplification (Chr. 5q). The blot below shows sample 7, a tumor not associated with radiation, demonstrating a loss of *CDKN2A* (Chr. 9p).

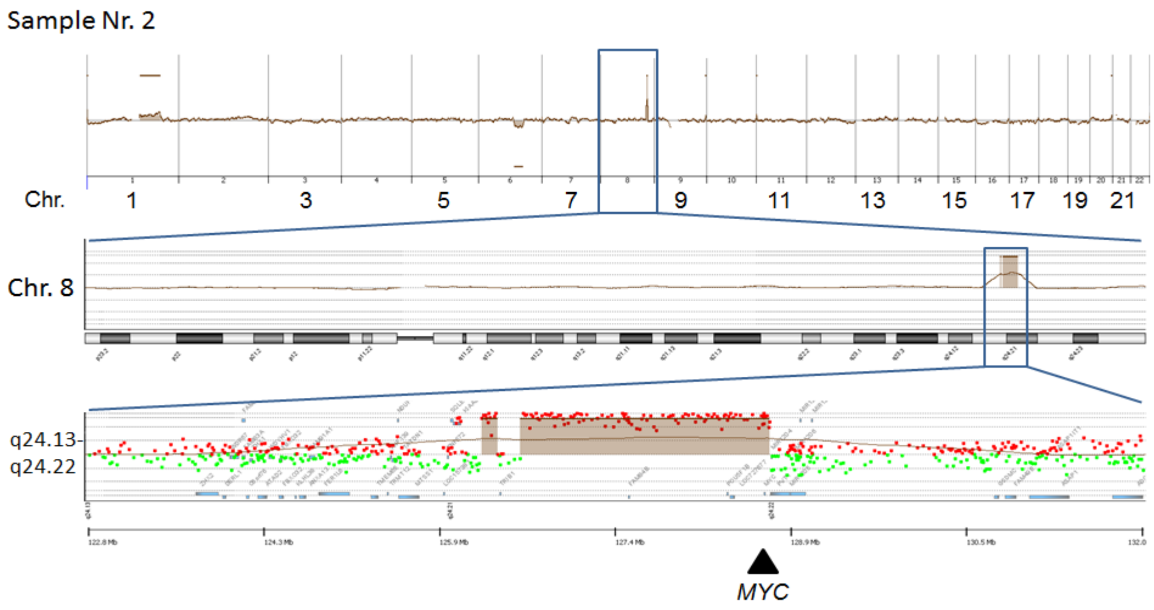

Supplemental Figure 2. Detailed representation of a high level *MYC* amplification. Representative whole genome copy number alteration blot of sample 2 analyzed by Agilent 180k array. The tumor is known to have arisen post-irradiation and demonstrates a high level amplification on Chromosome 8q containing the *MYC* locus. A detailed view of the amplification involving the *MYC* locus is shown in the lower part of the figure.
